# Fluoride Exposure from Drinking Water Increases the Risk of Stroke: An Ecological Study in Changwu Town, China

**DOI:** 10.3390/toxics12090679

**Published:** 2024-09-18

**Authors:** Lin Yuan, Hongna Sun, Yue Li, Zhifeng Xing, Shihui Yin, Fengyu Xie, Jing Zhou, Shuang Li, Liaowei Wu, Wei Huang, Teng Wang, Yanhui Gao, Lijun Zhao, Dianjun Sun

**Affiliations:** 1 Center for Endemic Disease Control, Chinese Center for Disease Control and Prevention, Harbin Medical University, Harbin 150081, China; 2 Key Lab of Etiology and Epidemiology, Education Bureau of Heilongjiang Province & Ministry of Health of P. R. China, Harbin Medical University, Harbin 150081, China; 3 Heilongjiang Provincial Key Lab of Trace Elements and Human Health Harbin Medical University, Harbin 150081, China; 4 Zhaodong City Center for Disease Control and Prevention, Zhaodong 151100, China; 5 Heilongjiang Provincial Center for Disease Control and Prevention, Harbin 150030, China; 6 Shaanxi Provincial People’s Hospital, Xi’an 712038, China; 7 Chongqing Municipal Center for Disease Control and Prevention, Chongqing 400707, China; 8 Beilun District People’s Hospital, Ningbo 315800, China

**Keywords:** endemic fluorosis, water fluoride, stroke prevalence rate, stroke mortality rate

## Abstract

Background: Stroke is a major cause of death globally and the leading cause in China. Excessive fluoride exposure has been linked to cardiovascular conditions related to stroke risk factors such as hypertension, atherosclerosis, dyslipidemia, and cardiomyopathy. However, evidence supporting the association between fluoride exposure and stroke risk is limited. Methods: We constructed an ecological study in Changwu Town, Heilongjiang Province, China, a typical endemic fluorosis area caused by excessive fluoride exposure from drinking water. We collected demographic data, stroke prevalence, and mortality information from 2017 to 2021. Fluoride exposure data were obtained from the national monitoring project on endemic fluorosis. Water fluoride concentrations were measured using the standardized methods. Trend changes in stroke rates were assessed using annual percentage change (APC). Differences in stroke rates among fluoride exposure groups were analyzed using chi-square tests. Results: From 2017 to 2021, the all-ages and age-standardized stroke prevalence rates of permanent residents in Changwu Town increased year by year, while the all-ages and age-standardized mortality rates did not change significantly. The prevalence rates of stroke were significantly higher in endemic fluorosis areas compared to non-endemic areas (*p* < 0.001). Stratifying the population into tertile groups based on the water fluoride cumulative exposure index (WFCEI) revealed statistically significant differences in stroke prevalence rates (*p* < 0.001), showing a dose–response relationship with the WFCEI. However, the all-ages and age-standardized mortality rates of stroke were not found to be related to fluoride exposure. Conclusions: Long-term excessive fluoride exposure from drinking water may increase the risk of stroke prevalence, indicating fluoride overexposure as a potential risk factor for stroke.

## 1. Introduction

Stroke is a sudden onset of cerebral blood circulation disorder, which is the second leading cause of global death and the leading cause of death in China [1,2]. Established risk factors for stroke include age, hypertension, atherosclerosis, dyslipidemia, obesity, smoking, physical inactivity, etc. [3]. In addition to these well-known risk factors, emerging epidemiological investigations have highlighted the potential role of environmental pollutants as novel risk factors for stroke and other cardiovascular diseases [4,5]. For example, prospective population cohort studies have confirmed that long-term exposure to PM2.5 pollution significantly increases the risk of stroke in the Chinese population [6].

Fluorine, an extremely active non-metallic element, is widely present in the environment in the form of various chemical compounds found in air, water, and soil [7]. Excessive fluoride exposure is known to cause fluorosis, with dental and skeletal fluorosis being the most recognizable clinical manifestations [8]. Recent studies have demonstrated that fluoride exposure can not only impact skeletal health but also lead to a range of non-skeletal injuries affecting the cardiovascular, nervous, and immune system [9,10,11,12]. Currently, public health is increasingly focused on researching fluoride-induced non-skeletal damage and its underlying mechanism [13,14], characterized by accumulation, long-term effects, and complexity [15,16]. Overexposure to fluoride through drinking water is a global public health concern, impacting billions of people worldwide [17]. In the past forty years, extensive water improvement and fluoride reduction initiatives have been implemented in most endemic fluorosis areas in China where the fluoride concentration in drinking water exceeded 1.2 mg/L [18]. However, there are still areas in China where high fluoride levels in water have not been addressed, or where the fluoride levels remain above standards even after improvement efforts, posing critical safety challenges [19,20].

Epidemiological and experimental studies evidence suggests fluoride’s potential neurotoxicity and cardiovascular effects [21,22,23], including its ability to breach the blood–brain barrier [24]. Fluoride is intricately linked to stroke risk factors such as hypertension, atherosclerosis, dyslipidemia, hyperglycemia, and cardiomyopathy [9,25,26,27,28,29,30,31]. However, research on chronic fluoride exposure and strokes remains limited. Investigating the relationship is crucial for public health, particularly in nations like China, where residents face health threats from both endemic fluorosis and stroke.

This study conducts an ecological investigation in Changwu Town, a representative endemic fluorosis area in Northeast China, to analyze the epidemiological patterns and trends in stroke among permanent residents from 2017 to 2021. By contrasting the prevalence and mortality rates of stroke across different levels of fluoride exposure, the aim is to elucidate the association between fluoride exposure and stroke risk. The findings seek to offer a scientific foundation for the comprehensive prevention and control of endemic fluorosis and stroke.

## 2. Materials and Methods

### 2.1. Study Location and Population

**Figure 1:**
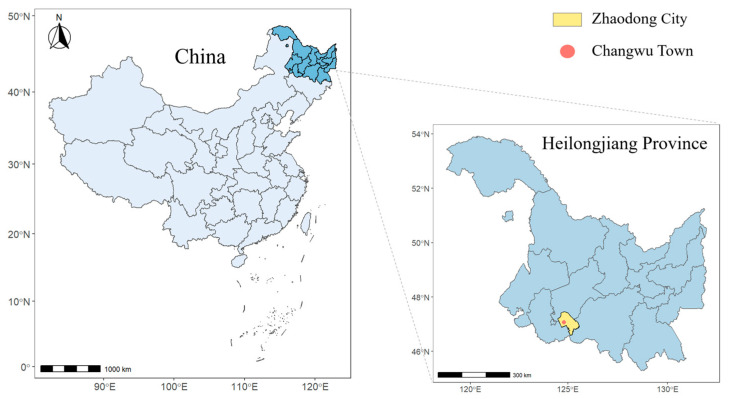
Specific location of the survey site in China.

This study was conducted in Changwu Town, a rural area in Zhaodong City, Heilongjiang Province, in 2022. Changwu Town, situated in the northern part of Zhaodong City, was once a place seriously affected by the drinking water type of endemic fluorosis (Figure 1). It governs 77 villages, with a permanent population of around 22,000. Among these, 44 villages were affected by endemic fluorosis due to drinking water. This study was conducted on each village as a unit, and all permanent residents of each village were included.

### 2.2. Stroke Information Collection

This study designed questionnaires that encompassed the following: (1) basic information, including village names, total permanent resident numbers, gender distribution, and age group demographics annually from 2017 to 2021; (2) data on the prevalence and mortality of stroke among all permanent residents in each village over a five-year period. The investigation was conducted by village doctors who conducted household visits to personally interview residents and complete the questionnaires. These investigators received standardized training by professionals at the Center for Endemic Disease Control (CEDC), Chinese Center for Disease Control and Prevention. Diagnosis of stroke cases and fatalities was performed by doctors from township healthcare centers or higher-level medical facilities. Additionally, data on stroke-related deaths were obtained from the Death Registration System, provided by the Center for Disease Control and Prevention of Zhaodong City. This data collection method served as a complementary source alongside face-to-face interviews.

### 2.3. Data Sources of Historical Water Fluoride Exposure

Based on the national monitoring data of the drinking water type of endemic fluorosis in 2021 from the CEDC, we obtained detailed information on the fluoride exposure from drinking water for the study subjects, including the historical water fluoride levels, the year of water improvement, the operation status of water improvement projects, and the current water fluoride levels across all villages. According to the “Classification of Endemic Fluorosis Areas” (GB 17018-2011) standard, a village is determined as a drinking water type of endemic fluorosis area if the fluoride concentration in the drinking water exceeds 1.2 mg/L and the prevalence of dental fluorosis among local children aged 8–12 surpasses 30%.

### 2.4. Water Sample Collection

Centralized water supplies are provided for villages in Changwu Town. A total of 15 mL of terminal water sample was collected for each project using clean polyethylene bottles.

### 2.5. Determination of Fluoride Concentrations in Water Sample

The fluoride concentrations of water samples were analyzed using the ion-selective electrode method (WS/T 89-2015, Industry standard of the People’s Republic of China). All reference solutions were deionized water and all chemicals used in the tests were of analytical purity. Standards were prepared from a 1000 μg/mL F^−^ stock solution (GSB 04-1771-2004). Standard fluoride solutions with concentrations of 1.0 mg/L and 10.0 mg/L were used to make a standard curve series. The standard curve graph for F^−^ was obtained with the calibration solution range of 0.1, 0.2, 0.5, 1.0, 2.0, 5.0, and 10.0 mg/L. Before measurement, 5 mL of total ionic strength adjustment buffer and 5 mL of the water sample were added to each test cup, and then a fluorine-ion-selected electrode and a reference electrode were connected to a fluoride ion meter (all instruments are from Shanghai Instrument Science Co., Ltd., Shanghai, China). The standard calibration curve was plotted from the absorbance values obtained from the seven standards of fluoride solutions by taking Log concentration (mg/L) on the *x*-axis and electric potential values on the *y*-axis (Figure S1A). The linear regression equation was y = −60.104x + 268.7, and R^2^ = 1.000. The Limit of Detection (LOD) was 0.1 mg/L. Two quality control samples (GSB 07-1194-2000 201758 and 201760) accorded with the credible ranges of the standard value. Each sample was measured two times, and then the average fluoride concentration (mg/L) was calculated.

### 2.6. Water Fluoride Cumulative Exposure Index (WFCEI)

For this study, the water fluoride cumulative exposure index (WFCEI) was developed to assess the cumulative fluoride exposure dose for each village. It was calculated as follows: (year of investigation − year of water improvement) × fluoride concentration in current drinking water + [average age of study population − (year of investigation − year of water improvement)] × fluoride concentration in historical drinking water.

### 2.7. Statistical Analysis

The calculation method for stroke prevalence and mortality rates was to divide the annual numbers of stroke patients and deaths by the annual population, respectively. Using the population composition of the 7th National Population Census of China in 2021 as the standard, age standardization was applied to calculate the age-standardized prevalence and mortality rates. We stratified the data of stroke prevalence and mortality rates in Changwu Town by gender, age (≤34, 35–44, 45–54, 55–64, 65–74, and ≥75 years old), disease type (ischemic and hemorrhagic stroke), historical water fluoride (≤1.2 and >1.2 mg/L, e.g., endemic and non-endemic fluorosis areas), concentration of current water fluoride (CWF) (≤1 and >1 mg/L), water improvement period (non-endemic areas, 1~5, 6~10, 11~15, and 16~20 years), and WFCEI (Tertile 1, Tertile 2, and Tertile 3).

The data were analyzed using IBM SPSS Statistics Version 23.0 (SPSS, Inc., Chicago, IL, USA) and Joinpoint software, version 4.9.1.0 (US Statistical Research and Applications Branch, National Cancer Institute, Rockville, MD, USA). The chi-square test was used for inter-group comparison of rates. The trend change in the rates was described using the annual percentage change (APC); the APC was 100{exp(β) − 1}, and β was the estimated regression coefficients. The exponential distribution regression model was used to estimate the value of β, and the t-test was used to test the APC. Hypothesis testing for all analyses was based on two-tailed rejection regions, and a *p*-value < 0.05 was applied to declare statistical significance.

## 3. Results

### 3.1. Population and Fluoride Exposure in Changwu Town

**Figure 2:**
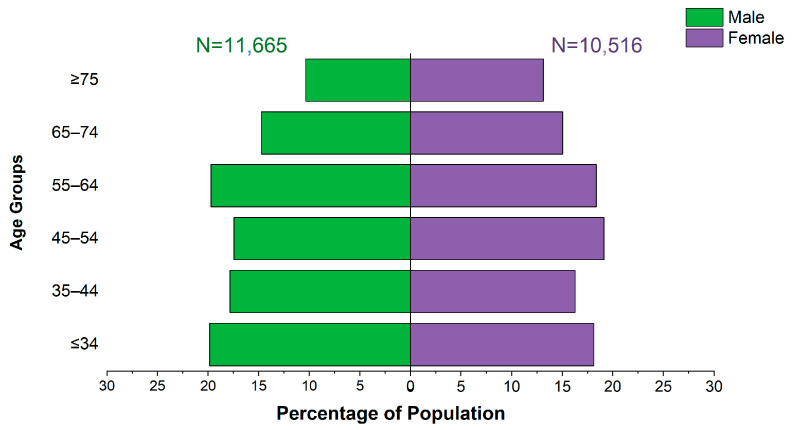
Demographic composition of residents in Changwu Town (2021).

The fundamental data and demographic composition of the permanent population in Changwu Town from 2017 to 2021 are detailed in Table S1 and depicted in Figure 2. In 2021, the total permanent population of Changwu Town amounted to 22,181 individuals, comprising 11,665 males (52.59%) and 10,516 females (47.41%).

Monitoring data showed that 44 villages (57.14%) in Changwu Town were classified as drinking water type of endemic fluorosis areas, encompassing 10,356 residents (46.69%). Within these endemic areas, historical water fluoride concentrations ranged from 1.2 to 4.5 mg/L, with water improvement initiatives spanning from 2006 to 2016, on average 10.93 ± 3.60 years. Following the water improvement project, all endemic villages received upgraded water sources, lowering fluoride concentrations to below 1.2 mg/L. During the study, 38 water samples were collected on site, and laboratory analyses revealed fluoride concentrations ranging from 0.14 to 1.25 mg/L, aligning with the monitoring data. The median concentration of CWF across the 77 villages in Changwu Town was 0.87 mg/L (with an interquartile range of 0.49–1.02 mg/L), averaging 0.76 ± 0.31 mg/L (Figure S1B). The characteristics of fluoride exposure from drinking water in this study and similar studies in other locations worldwide are shown in Table 1.

### 3.2. Descriptive Analysis of the Prevalence and Mortality Rates of Stroke in Changwu Town from 2017 to 2021

The basic data of stroke patients and deaths in Changwu Town from 2017 to 2021 are shown in Table S2. Over this period, the age-standardized prevalence rates of stroke exhibited an increasing trend: 1450.8, 1935.9, 2337.4, 2667.3, and 3024.5 per 100,000 (*p* = 0.003). The age-standardized mortality rates showed no significant variance across the years: 64.1, 87.4, 143.7, 142.5, and 65.9 per 100,000 (*p* = 0.630) (Table 2).

From 2017 to 2021, both all-ages and age-standardized prevalence rates of stroke in both genders escalated annually (all *p* < 0.01). While males exhibited higher age-standardized stroke prevalence and mortality rates than females, the differences were not statistically significant (Table S3 and Figure S2). Notable variations in prevalence and mortality rates were observed among diverse age groups (all *p* < 0.05), with the 55–64 age group registering the highest prevalence rate and those aged ≥75 displaying the highest mortality rate (Table S4 and Figure S3). Ischemic stroke prevalence rates significantly exceeded hemorrhagic stroke rates in both all-ages and age-standardized analyses from 2017 to 2021 (all *p* < 0.001). In 2019 and 2020, the all-ages and age-standardized mortality rates for ischemic stroke were significantly higher than those for hemorrhagic stroke (all *p* < 0.05) (Table S5 and Figure S4).

### 3.3. Comparative Analysis of Stroke Prevalence and Mortality Rates across Varying Fluoride Exposure Levels

The basic data of stroke patients and deaths under different levels of fluoride exposure in Changwu Town from 2017 to 2021 are shown in Table S6.

#### 3.3.1. Comparison of Stroke between Endemic and Non-Endemic Fluorosis Areas

**Figure 3:**
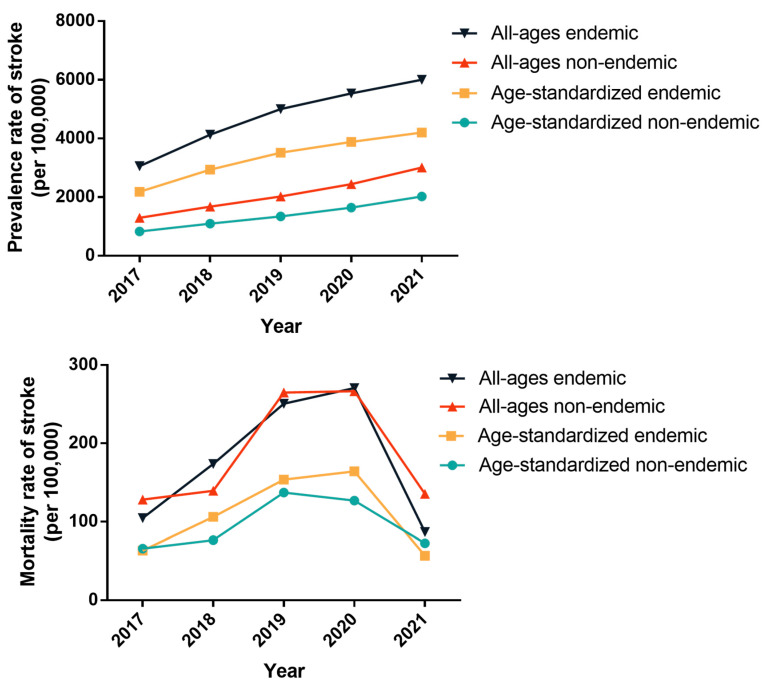
The prevalence and mortality rates of stroke in endemic and non-endemic fluorosis areas in Changwu Town from 2017 to 2021 (per 100,000).

From 2017 to 2021, both all-ages and age-standardized prevalence rates of stroke in both endemic and non-endemic fluorosis areas exhibited yearly increases (all *p* < 0.05), with significantly higher rates in residents living in endemic areas than in non-endemic areas each year (all *p* < 0.001). There were no significant trends in the change in mortality rates of stroke in both endemic and non-endemic areas, and also there were no statistically significant differences in all-ages mortality rates between the two groups. Post age-standardization, while mortality rates in endemic areas were higher than those in non-endemic areas from 2018 to 2020, these differences remained statistically insignificant (Table S7 and Figure 3).

Upon gender stratification, both all-ages and age-standardized prevalence rates of stroke for males and females in endemic areas were still statistically higher than those in non-endemic areas (all *p* < 0.001) (Table S8 and Figure S5). There were still no statistically significant differences in stroke mortality rates between endemic and non-endemic areas when analyzed by gender (Table S9 and Figure S6).

#### 3.3.2. Comparison of Stroke across Different Current Water Fluoride (CWF) Groups

**Figure 4:**
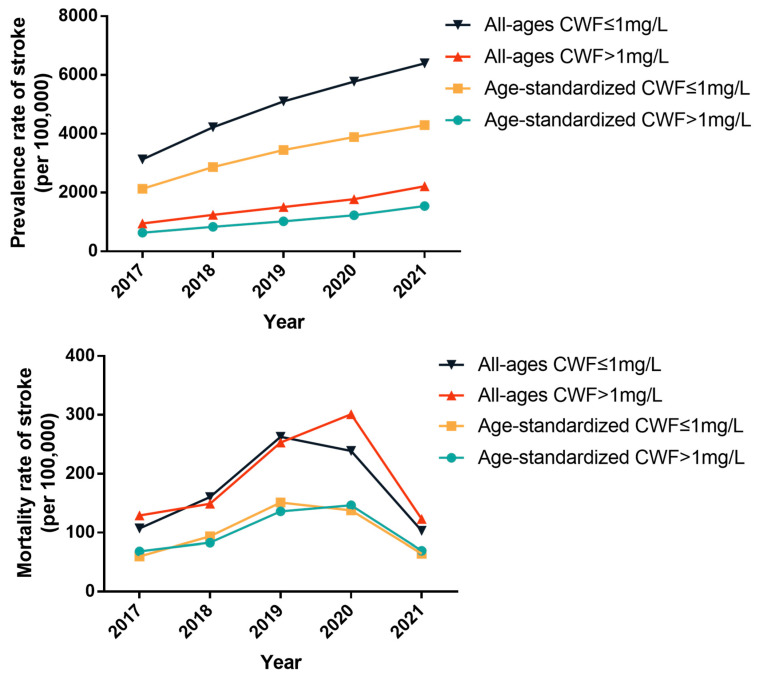
The prevalence and mortality rates of stroke in different current water fluoride (CWF) groups in Changwu Town from 2017 to 2021 (per 100,000).

CWF was categorized into two groups using a 1 mg/L cut-off value, with concentration ranges of 0.14–0.99 mg/L and 1.01–1.25 mg/L, respectively. From 2017 to 2021, both all-ages and age-standardized prevalence rates of stroke significantly increased in both groups (all *p* < 0.01). The all-ages and age-standardized prevalence rates of stroke among residents living in CWF ≤ 1 mg/L areas were significantly higher than those living in CWF > 1 mg/L areas each year (all *p* < 0.001). However, there were no significant changes in mortality rates over the five-year period or between the two groups (Table S10 and Figure 4).

#### 3.3.3. Comparison of Stroke among Different Water Improvement Periods

**Figure 5:**
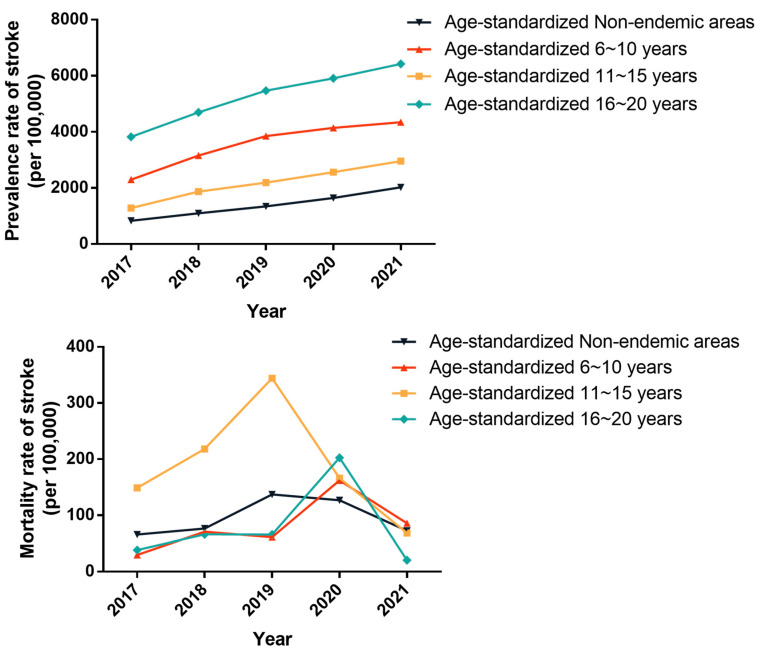
The prevalence and mortality rates of stroke in groups with different water improvement periods in Changwu Town from 2017 to 2021 (per 100,000).

Residents were divided into five groups based on the water improvement period: non-endemic areas, 1–5 years, 6–10 years, 11–15 years, and 16–20 years. Since all water improvement period in Changwu Town exceeded 5 years, only four groups of data were analyzed. From 2017 to 2021, the all-ages and age-standardized prevalence rates of stroke in the four groups exhibited an annual increase (all *p* < 0.01), with statistically significant differences among the groups (all *p* < 0.001). The non-endemic areas showed the lowest all-ages and age-standardized prevalence rates, followed by the 11–15 years, 6–10 years, and 16–20 years water improvement groups in that order. There were no significant changes in the all-ages mortality rates of stroke over the five-year period. In 2019, there was a statistically significant difference in all-ages and age-standardized mortality rates among the four groups with different water improvement periods (all *p* < 0.01), and the rates of the group where water had been improved for 11–15 years were the highest. Apart from 2019, there were no statistically significant differences in all-ages and age-standardized mortality rates among the four groups (*p* > 0.05) (Table S11 and Figure 5).

#### 3.3.4. Comparison of Stroke among Groups with Different Water Fluoride Cumulative Exposure Index (WFCEI)

**Figure 6:**
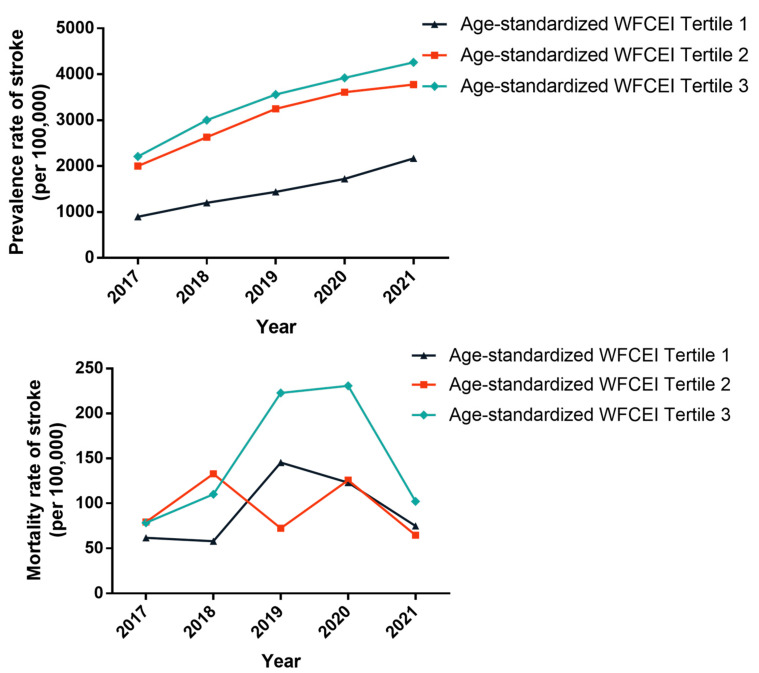
The prevalence and mortality rates of stroke in different WFCEI groups in Changwu Town from 2017 to 2021 (per 100,000).

To assess the cumulative fluoride exposure, the WFCEI was further calculated, ranging from 7.00 to 199.38 across 77 villages. These values were divided into three groups based on tertiles: the Tertile 1 group (WFCEI 7.00–51.00), Tertile 2 group (WFCEI 51.53–74.61), and Tertile 3 group (WFCEI 74.93–199.38). From 2017 to 2021, the all-ages and age-standardized prevalence rates of stroke in the three groups displayed annual increases (all *p* < 0.05), showing a dose–response trend with a higher WFCEI associated with higher prevalence rates. The highest prevalence rates were observed in the Tertile 3 group, while the lowest rates were in the Tertile 1 group, with statistically significant differences among the three WFCEI groups (all *p* < 0.001). There were no significant changes in mortality rates over the five-year period, and the differences in mortality rates among the different WFCEI groups were also not statistically significant (Table S12 and Figure 6).

## 4. Discussion

Our study found that the all-ages and age-standardized prevalence rates of stroke in Changwu Town increased steadily from 2017 to 2021, which was consistent with the trend of total China [32]. A study showed that the age-standardized prevalence rate of stroke in China in 2019 was 1468.9 per 100,000, and that in Heilongjiang it was 2049.5 per 100,000, which was the highest level in China [32,33]. The standardized prevalence rate of stroke in Changwu Town in 2019 was 2337.4 per 100,000, higher than the national level and even higher than the provincial level. According to the Brief Report on Stroke Prevention and Treatment in China in 2021, the all-ages mortality rates of stroke increased by 7.4% from 2010 to 2019 [34]. The age-standardized mortality rates of stroke decreased by 39.8% from 1990 to 2019 [32]. However, our study did not observe a significant trend in the all-ages and standardized mortality rates of stroke among residents in Changwu Town over the five-year period. The mortality rates gradually increased from 2017 to 2020, with an especially significant increase in 2019 and 2020, but sharply decreased in 2021. With the pandemic of Corona Virus Disease 2019 (COVID-19) in 2020–2022 in China, becoming especially serious in 2021 and 2022 in Changwu Town, the residents tended to seek medical treatment and died more as a result of respiratory symptoms [35], which may partly explain the sharp decrease in the mortality rate of stroke in 2021. In 2019, the all-ages mortality rate of stroke in Changwu Town (258.3 per 100,000) was much higher than in rural China (158.6 per 100,000); also, the standardized mortality rate in Changwu Town (143.7 per 100,000) was higher than in total China (127.2 per 100,000) [34]. Changwu Town, a rural area in Northeast China, is bound to face plights like the outflow of young adults and aging of the population, which combined with special diet habits, lifestyle, and the cold climate may contribute to the higher stroke burden of local residents [36,37]. The stroke prevention and control of rural residents in Northeast China should be paid enough attention.

From 2017 to 2021, the all-ages and standardized prevalence rates of stroke in both sexes in Changwu Town increased gradually, with higher rates in men than in women, which was consistent with other reports [32,33]. There was a statistically significant difference in the prevalence rates of stroke among different age groups, with the highest rates observed in the 55–64 years group. It was reported that the prevalence rates of stroke in China increased with age, reaching the highest level between 70 and 79 years old [29], but the peak age of stroke in our study was younger, which may be attributed to the rising trend in stroke incidence in the younger population [38,39] and the high level of stroke mortality in the elderly population in Changwu Town. The result of standardized prevalence rates in 2019 showed that ischemic stroke in Changwu Town was higher than the national level (2207.0 vs. 1256 per 100,000), while hemorrhagic stroke in Changwu was lower than the national level (175.3 vs. 215 per 100,000) [40]. Therefore, we need to emphasize the prevention of stroke incidence, especially ischemic stroke, in Changwu Town.

Measures to improve water quality and reduce fluoride content in drinking water type of endemic fluorosis areas have been implemented in China for more than half a century. But in some places, the residents are still threatened by high-level fluoride exposure from drinking water due to the abnormal operation of the water improvement projects, the rebound of fluoride concentration, and the lack of low-fluoride water sources [19,20]. Changwu Town is a typical drinking water type of endemic fluorosis area in China, formed by high fluorine exposure from shallow-bed groundwater. The water improvement period for Changwu Town was relatively short, and the time of fluoride exposure was relatively long. Comprehensive understanding of the health effects of fluoride exposure is crucial for the assessment of health risks for populations in countries and regions with fluorosis and for making relevant health policies.

The classification of endemic and non-endemic areas reflected the historical exposure to water fluoride. The result of this study showed the all-ages and age-standardized prevalence rates of stroke in endemic areas were significantly higher than in non-endemic areas and there were no statistically significant differences in mortality rates of stroke between the two groups, indicating that historical exposure to high fluoride concentrations in drinking water increased the risk of stroke. Although the drinking water of Changwu Town had been improved for nearly 11 years on average, the risk of stroke in endemic villages was still particularly high. Fluoride is a cumulative environmental toxin that has long-term effects on human health [18,19]. A case–control study conducted in Xinjiang, China, concluded that a high level of fluoride in drinking water can cause pulmonary function damage in skeletal fluorosis patients even after water improvement for 18 years [41]. The health repercussions stemming from excessive fluoride exposure may not be entirely reversible, underscoring the substantial importance of prevention and control measures for fluorosis. On the other side, there is a linear increase in stroke prevalence despite the improvement in water quality, which indicates that the risk factors of stroke, such as special diet habits and lifestyle, together with population aging may play a more decisive role here than fluoride exposure.

The concentration of CWF reflected the recent exposure to water fluoride after water improvement. This study also found that CWF was associated with stroke prevalence rates, but had no effect on stroke mortality rates. In Changwu Town, the all-ages and age-standardized prevalence rates of stroke in villages with CWF ≤ 1 mg/L were significantly higher than in areas with CWF > 1 mg/L. However, it is important to note that this does not necessarily imply that lower fluoride levels lead to a higher risk of stroke. In Changwu Town, endemic areas, where historical water fluoride levels exceeded 1.2 mg/L, were successively provided with improved water based on the principle of ‘severe priority and then slight’. In this study, 76.0% (38/50) villages with CWF ≤ 1 mg/L (rang from 0.14 to 0.99 mg/L) belonged to historical endemic areas with historical water fluoride exceeding 1.2 mg/L, while 77.8% (21/27) villages with CWF > 1 mg/L (rang from 1.01 to 1.25 mg/L) were located in non-endemic areas. Relying solely on current fluoride exposure for investigation and analysis can lead to erroneous conclusions. Similarly, the prevalence rates of stroke in Changwu Town did not decrease with an extended period of water improvement. The highest prevalence rates were observed in the group where water had been improved for 16–20 years because the villages in this group were previously severely affected by fluorosis.

There are significant differences in the historical water fluoride concentrations, CWF, and water improvement period among different villages in Changwu Town. Evaluating long-term exposure accumulation using a single indicator is unreasonable. The WFCEI integrates the historical water fluoride concentrations, CWF, water improvement period, and the age of population, providing a comprehensive evaluation of the total fluoride exposure from drinking water for residents in each village. The all-ages and standardized prevalence rates of stroke among residents in Changwu Town from 2017 to 2021 showed a dose–response increasing trend with the WFCEI, and the differences among the groups were statistically significant, which further indicated that fluoride exposure could increase the risk of stroke and there might be a dose–response relationship.

Collectively, these findings indicated that fluoride exposure may be a risk factor of stroke despite the improvement in water quality. The historical water fluoride concentrations in the endemic areas of our study ranged from 1.2 to 4.5 mg/L, which were similar to or a little lower than the studies concerning the effects of fluoride exposure on overweight, obesity and carotid atherosclerosis carried out in China [26,29], and on hypertension carried out in Iran [25]. The CWF concentration for the total study subjects in our study was between 0.14 and 1.25 mg/L, similar to the studies in Indonesia and the United States related to intelligence and attention deficit [21,22]. The current WHO recommended guideline for fluoride in drinking water is 1.5 mg/L, and that in China is 1.0 mg/L for large-scale centralized water engineering and 1.2 mg/L for small-scale engineering and decentralized water supply [19]. Since the implementation of fluoridation of the drinking water to populations has always been a controversial topic, the US Department of Health and Human Services revised the guidelines in 2015, lowering the recommended level from 0.7 to 1.2 mg/L fluoride to a standardized 0.7 mg/L F^−^ to balance the benefits of caries prevention with the risk of dental fluorosis [42]. A Malaysian study even showed that reducing the fluoride concentration in water from 0.7 to 0.5mg/L can reduce fluorosis and maintain the effect of preventing caries [43]. In our study, the water fluoride levels in the endemic areas exceed the recommended levels of fluoride in drinking water, and did not contradict public dental health recommendations. All the evidence indicated that excessive fluoride exposure, even a low to moderate degree of fluoride exposure, could lead to non-skeletal injuries of human beings.

Our study presented several strengths. We comprehensively described the epidemiological situation and trend in stroke in Changwu Town from 2017 to 2021, providing basic data for the prevention and control of stroke in the locality. For the first time, we reported the impact of fluoride exposure in drinking water on stroke based on multiple perspectives of water fluoride exposure evaluation, particularly with the design and application of the WFCEI. Our study provides epidemiological evidence and supports the hypothesis that excessive fluoride exposure could increase the risk of stroke.

This study was subject to some limitations. First, our study was an ecological study and was of incomplete adequacy as evidence to explain the stroke risk of fluoride exposure, and more longitudinal studies are needed. Second, this study is only a survey conducted in Changwu Town, Zhaodong City, a typical drinking water type of endemic fluorosis area in Heilongjiang Province, China. Therefore, extension of the conclusion to the entire population should be carried out with caution. Third, this study is an ecological study at the colony level, and there were many potential confounding factors of stroke such as sociodemography, environment, and lifestyle habits which might be difficult to control. Also, due to the epidemic of COVID-19, the data of stroke deaths may be biased.

## 5. Conclusions

In conclusion, our study suggests a correlation between long-term fluoride exposure and the risk of stroke prevalence. The findings indicate a consistent increase in stroke prevalence among all residents of Changwu Town from 2017 to 2021. Both all-ages and age-standardized stroke prevalence rates were notably higher in areas with endemic fluorosis compared to non-endemic regions, displaying a dose–response relationship with the WFCEI. Consequently, prolonged excessive fluoride exposure through drinking water may serve as a potential risk factor for stroke. These results could offer a theoretical basis for countries and regions affected by fluorosis to develop pertinent prevention and control strategies.

## Figures and Tables

**Table 1:** Characteristics of fluoride exposure from drinking water in Changwu Town and correlational studies.

Author (Year)	Study Design	Country	Fluoride Exposure from Drinking Water (mg/L)				Health Effect	Reference
			Variables	Range	Mean ± Standard Deviation	Median (Interquartile Range)		
Our study	Ecological study	China	CWF	0.14–1.25	0.76 ± 0.31	0.87 (0.49, 1.02)	Stroke	
			Historical water fluoride	0.14–4.50	1.51 ± 0.88	1.20 (1.02, 2.00)		
Yani, et al. (2021)	Cross-sectional study	Indonesia	CWF	0.30 and 1.60			Intelligence quotient (IQ)	[21]
Malin, et al. (2015)	Ecological study	the United States	CWF	0.70–1.20			Attention-deficit hyperactivity disorder (ADHD)	[22]
Dewey, et al. (2023)	Ecological cohort study	Canada	CWF	0–0.70			Poorer inhibitory control and cognitive flexibility	[23]
Yousefi, et al. (2018)	Cross-sectional study	Iran	CWF		0.68–10.30		Hypertension	[25]
Liu, et al. (2013)	Cross-sectional study	China	CWF	0.03–7.83	1.95 ± 1.13		Carotid atherosclerosis	[26]
Itai, et al. (2021)	Cross-sectional Study	Japan	CWF	0.02–0.15			Reduce insulin secretion and increase fasting plasma glucose levels	[28]
Liu, et al. (2019)	Cross-sectional study	China	CWF	0.20–3.50		1.00 (0.40, 1.53)	Overweight and obesity	[29]
Adali, et al. (2013)	Prospective study	Turkey	CWF		0.53 ± 0.06 and 2.74 ± 064		Impaired heart rate recovery	[30]

**Table 2:** The prevalence and mortality rates of stroke in Changwu Town from 2017 to 2021 (per 100,000).

Year	Number		Prevalence Rate		Mortality Rate	
	Patients	Deaths	All-Ages	Age-Standardized	All-Ages	Age-Standardized
2017	483	27	2101.6	1450.8	117.5	64.1
2018	633	35	2804.7	1935.9	155.1	87.4
2019	763	58	3398.3	2337.4	258.3	143.7
2020	866	60	3876.1	2667.3	268.6	142.5
2021	978	25	4409.3	3024.5	112.7	65.9
APC			18.9 **	18.7 **	9.5	7.6
APC 95%CI			12.1~26.2	11.9~26.0	−35.3~85.0	−34.5~76.7
*t*-value			9.3	9.2	0.5	0.5
*p*-value ^†^			**0.003**	**0.003**	0.622	0.671

Note: APC, annual percentage change. ^†^ *p*-value for APC comparison over five years. *p* < 0.05 is indicated in bold, ** *p* <0.01.

## Data Availability

The original contributions presented in the study are included in the article/Supplementary Materials, further inquiries can be directed to the corresponding author/s.

## Supplementary Materials

The following supporting information can be downloaded at: https://www.mdpi.com/article/10.3390/toxics12090679/s1, Table S1: Annual population of permanent residents in Changwu Town from 2017 to 2021; Figure S1: The detection and result of CWF concentration of 77 villages in Changwu Town. (A) The standard curve about the measured electric potential values and the Log concentration of CWF. (B) Histogram of CWF concentration of 77 villages in Changwu Town; Table S2: The numbers of stroke patients and deaths by sex, age, and disease type in Changwu Town from 2017 to 2021; Table S3: The prevalence and mortality rates of stroke by gender in Changwu Town from 2017 to 2021 (per 100,000); Figure S2: The prevalence and mortality rates of stroke by gender in Changwu Town from 2017 to 2021 (per 100,000); Table S4: The prevalence and mortality rates of stroke by age (years old) in Changwu Town from 2017 to 2021 (per 100,000); Figure S3: The prevalence and mortality rates of stroke by age in Changwu Town from 2017 to 2021 (per 100,000); Table S5: The prevalence and mortality rates of stroke by disease type in Changwu Town from 2017 to 2021 (per 100,000); Figure S4: The prevalence and mortality rates of stroke by disease type in Changwu Town from 2017 to 2021 (per 100,000); Table S6: The numbers of stroke patients and deaths under different levels of fluoride exposure in Changwu Town from 2017 to 2021; Table S7: The prevalence and mortality rates of stroke in endemic and non-endemic fluorosis areas in Changwu Town from 2017 to 2021 (per 100,000); Table S8: The all-ages and age-standardized prevalence rates of stroke by gender in endemic and non-endemic groups in Changwu Town from 2017 to 2021 (per 100,000); Figure S5: The all-ages and age-standardized prevalence rates of stroke by gender in endemic and non-endemic groups in Changwu Town from 2017 to 2021 (per 100,000); Table S9: The all-ages and age-standardized mortality rates of stroke by gender in endemic and non-endemic groups in Changwu Town from 2017 to 2021 (per 100,000); Figure S6: The all-ages and age-standardized mortality rates of stroke by gender in endemic and non-endemic groups in Changwu Town from 2017 to 2021 (per 100,000); Table S10: The prevalence and mortality rates of stroke in different current water fluoride (CWF) groups in Changwu Town from 2017 to 2021 (per 100,000); Table S11: The prevalence and mortality rates of stroke in groups with different water improvement periods (years) in Changwu Town from 2017 to 2021 (per 100,000); Table S12: The prevalence and mortality rates of stroke in different WFCEI groups in Changwu Town from 2017 to 2021 (per 100,000).

## Abbreviations

APC	annual percentage change
CEDC	Center for Endemic Disease Control
CWF	current water fluoride
WFCEI	water fluoride cumulative exposure index

## References

[1] Roth G.A., Mensah G.A., Johnson C.O., Addolorato G., Ammirati E., Baddour L.M., Barengo N.C., Beaton A.Z., Benjamin E.J., Benziger C.P. (2020). Global Burden of Cardiovascular Diseases and Risk Factors, 1990–2019: Update from the GBD 2019 Study. J. Am. Coll. Cardiol..

[2] Feigin V.L., Brainin M., Norrving B., Martins S., Sacco R.L., Hacke W., Fisher M., Pandian J., Lindsay P. (2022). World Stroke Organization (WSO): Global Stroke Fact Sheet 2022. Int. J. Stroke.

[3] Pandian J.D., Gall S.L., Kate M.P., Silva G.S., Akinyemi R.O., Ovbiagele B.I., Lavados P.M., Gandhi D.B.C., Thrift A.G. (2018). Prevention of stroke: A global perspective. Lancet.

[4] Ranta A., Ozturk S., Wasay M., Giroud M., Béjot Y., Reis J. (2023). Environmental factors and stroke: Risk and prevention. J. Neurol. Sci..

[5] Yankoty L.I., Gamache P., Plante C., Goudreau S., Blais C., Perron S., Fournier M., Ragettli M.S., Hatzopoulou M., Liu Y. (2022). Relationships between long-term residential exposure to total environmental noise and stroke incidence. Noise Health.

[6] Huang K., Liang F., Yang X., Liu F., Li J., Xiao Q., Chen J., Liu X., Cao J., Shen C. (2019). Long term exposure to ambient fine particulate matter and incidence of stroke: Prospective cohort study from the China-PAR project. BMJ.

[7] Singh G., Kumari B., Sinam G., Kriti Kumar N., Mallick S. (2018). Fluoride distribution and contamination in the water, soil and plants continuum and its remedial technologies, an Indian perspective—A review. Environ. Pollut..

[8] Dhar V., Bhatnagar M. (2009). Physiology and toxicity of fluoride. Indian J. Dent. Res..

[9] Davoudi M., Barjasteh-Askari F., Sarmadi M., Ghorbani M., Yaseri M., Bazrafshan E., Mahvi A.H., Moohebati M. (2021). Relationship of fluoride in drinking water with blood pressure and essential hypertension prevalence: A systematic review and meta-analysis. Int. Arch. Occup. Environ. Health.

[10] Ottappilakkil H., Babu S., Balasubramanian S., Manoharan S., Perumal E. (2023). Fluoride Induced Neurobehavioral Impairments in Experimental Animals: A Brief Review. Biol. Trace Elem. Res..

[11] Zhang C., Wang Y., Huang F., Zhang Y., Liu Y., Wang Q., Zhang X., Li B., Angwa L., Jiang Y. (2023). Fluoride induced metabolic disorder of endothelial cells. Toxicology.

[12] Du C., Xiao P., Gao S., Chen S., Chen B., Huang W., Zhao C. (2022). High Fluoride Ingestion Impairs Bone Fracture Healing by Attenuating M2 Macrophage Differentiation. Front. Bioeng. Biotechnol..

[13] Wu S., Wang Y., Iqbal M., Mehmood K., Li Y., Tang Z., Zhang H. (2022). Challenges of fluoride pollution in environment: Mechanisms and pathological significance of toxicity—A review. Environ. Pollut..

[14] Zhou J., Sun D., Wei W. (2023). Necessity to Pay Attention to the Effects of Low Fluoride on Human Health: An Overview of Skeletal and Non-skeletal Damages in Epidemiologic Investigations and Laboratory Studies. Biol. Trace Elem. Res..

[15] Nakamoto T., Rawls H.R. (2018). Fluoride Exposure in Early Life as the Possible Root Cause of Disease in Later Life. J. Clin. Pediatr. Dent..

[16] Chachra D., Limeback H., Willett T.L., Grynpas M.D. (2010). The long-term effects of water fluoridation on the human skeleton. J. Dent. Res..

[17] Podgorski J., Berg M. (2022). Global analysis and prediction of fluoride in groundwater. Nat. Commun..

[18] Wang F., Li Y., Tang D., Zhao J., Yang X., Liu Y., Peng F., Shu L., Wang J., He Z. (2021). Effects of water improvement and defluoridation on fluorosis-endemic areas in China: A meta-analysis. Environ. Pollut..

[19] Wang C., Gao Y., Wang W., Zhao L., Zhang W., Han H., Shi Y., Yu G., Sun D. (2012). A national cross-sectional study on effects of fluoride-safe water supply on the prevalence of fluorosis in China. BMJ Open.

[20] Wu J. (2020). Challenges for Safe and Healthy Drinking Water in China. Curr. Environ. Health Rep..

[21] Yani S.I., Seweng A., Mallongi A., Nur R., Abdullah M.T., Salmah U., Sirajuddin S., Basir-Cyio M., Mahfudz, Anshary A. (2021). The influence of fluoride in drinking water on the incidence of fluorosis and intelligence of elementary school students in Palu City. Gac. Sanit..

[22] Malin A.J., Till C. (2015). Exposure to fluoridated water and attention deficit hyperactivity disorder prevalence among children and adolescents in the United States: An ecological association. Environ. Health.

[23] Dewey D., England-Mason G., Ntanda H., Deane A.J., Jain M., Barnieh N., Giesbrecht G.F., Letourneau N. (2023). Fluoride exposure during pregnancy from a community water supply is associated with executive function in preschool children: A prospective ecological cohort study. Sci. Total Environ..

[24] Strazielle N., Ghersi-Egea J.F. (2013). Physiology of blood-brain interfaces in relation to brain disposition of small compounds and macromolecules. Mol. Pharm..

[25] Yousefi M., Yaseri M., Nabizadeh R., Hooshmand E., Jalilzadeh M., Mahvi A.H., Mohammadi A.A. (2018). Association of Hypertension, Body Mass Index, and Waist Circumference with Fluoride Intake; Water Drinking in Residents of Fluoride Endemic Areas, Iran. Biol. Trace Elem. Res..

[26] Liu H., Gao Y., Sun L., Li M., Li B., Sun D. (2013). Assessment of relationship on excess fluoride intake from drinking water and carotid atherosclerosis development in adults in fluoride endemic areas, China. Int. J. Hyg. Environ. Health.

[27] Pérez-Maldonado I.N., De la Trinidad-Chacón C.G., Perez-Lopez A.L., Perez-Lopez A.A. (2023). Association between urinary fluoride concentrations and the prevalence of metabolic syndrome in adult individuals from the Central Region of Mexico. Int. J. Environ. Health Res..

[28] Itai K., Onoda T., Nohara M., Kuribayashi T., Tanno K., Ohsawa M., Mori M., Okayama A. (2021). Slightly Elevated Serum Ionic Fluoride Levels Inhibit Insulin Secretion and Increase Glucose Levels in a General Japanese Population: A Cross-sectional Study. Biol. Trace Elem. Res..

[29] Liu L., Wang M., Li Y., Liu H., Hou C., Zeng Q., Li P., Zhao Q., Dong L., Yu X. (2019). Low-to-moderate fluoride exposure in relation to overweight and obesity among school-age children in China. Ecotoxicol. Environ. Saf..

[30] Adali M.K., Varol E., Aksoy F., Icli A., Ersoy I.H., Ozaydin M., Erdogan D., Dogan A. (2013). Impaired heart rate recovery in patients with endemic fluorosis. Biol. Trace Elem. Res..

[31] Quadri J.A., Sarwar S., Pinky Kar P., Singh S., Mallick S.R., Arava S., Nag T.C., Roy T.S., Shariff A. (2018). Fluoride induced tissue hypercalcemia, IL-17 mediated inflammation and apoptosis lead to cardiomyopathy: Ultrastructural and biochemical findings. Toxicology.

[32] Ma Q., Li R., Wang L., Yin P., Wang Y., Yan C., Ren Y., Qian Z., Vaughn M.G., McMillin S.E. (2021). Temporal trend and attributable risk factors of stroke burden in China, 1990-2019: An analysis for the Global Burden of Disease Study 2019. Lancet Public Health.

[33] Wang W., Jiang B., Sun H., Ru X., Sun D., Wang L., Wang L., Jiang Y., Li Y., Wang Y. (2017). Prevalence, Incidence, and Mortality of Stroke in China: Results from a Nationwide Population-Based Survey of 480 687 Adults. Circulation.

[34] Report on Stroke Prevention and Treatment in China Writing Group (2023). Brief report on stroke prevention and treatment in China (2021). Chin. J. Cerebrovasc. Dis..

[35] Melnick G., O’Leary J.F., Zaniello B.A., Abrishamian L. (2022). COVID-19 driven decline in emergency visits: Has it continued, is it permanent, and what does it mean for emergency physicians?. Am. J. Emerg. Med..

[36] Zhang F.L., Guo Z.N., Wu Y.H., Liu H.Y., Luo Y., Sun M.S., Xing Y.Q., Yang Y. (2017). Prevalence of stroke and associated risk factors: A population based cross sectional study from northeast China. BMJ Open.

[37] Chen C., Li J., Huang J. (2022). Spatial-Temporal Patterns of Population Aging in Rural China. Int. J. Environ. Res. Public Health.

[38] Putaala J. (2020). Ischemic Stroke in Young Adults. Continuum.

[39] Vrudhula A., Zhao J., Liu R. (2019). Too Young to Have a Stroke?—A Global Health Crisis. Stroke Vasc. Neurol..

[40] Report on Stroke Prevention and Treatment in China Writing Group (2022). Brief report on stroke prevention and treatment in China (2020). Chin. J. Cerebrovasc. Dis..

[41] Zhang J., Dili X., Ji F., Liu K. (2008). An Investigation on the Pulmonary Function of Skeletal Fluorosis Patients in Water -improving Fluorosis Region. Chin. J. Ctrl. Enden. Dis..

[42] Veneri F., Vinceti S.R., Filippini T. (2024). Fluoride and caries prevention: A scoping review of public health policies. Ann. Ig..

[43] Mohd Nor N.A., Chadwick B.L., Farnell D.J.J., Chestnutt I.G. (2021). Factors associated with dental fluorosis among Malaysian children exposed to different fluoride concentrations in the public water supply. J. Public Health Dent..

