# Supplementary material for: Fluoride Exposure from Drinking Water Increases the Risk of Stroke: An Ecological Study in Changwu Town, China
Source: Toxics. 2024 Sep 18;12(9):679. doi: 10.3390/toxics12090679 (PMC11436047; doi:10.3390/toxics12090679)
Supplement: Supplementary file 1 [file toxics-12-00679-s001.zip › toxics-3190919-supplementary.pdf]

Supplementary material

Fluoride Exposure from Drinking Water Increases the Risk of Stroke: An Ecological Study in Changwu Town, China

List of Tables and Figures

Table S1. Annual population of permanent residents in Changwu Town from 2017 to 2021.

Figure S1. The detection and result of CWF concentration of 77 villages in Changwu Town. (A) The standard curve about the measured electric potential values and the Log concentration of CWF. (B) Histogram of CWF concentration of 77 villages in Changwu Town.

Table S2. The numbers of stroke patients and deaths by sex, age and disease types in Changwu Town from 2017 to 2021.

Table S3. The prevalence and mortality rates of stroke by gender in Changwu Town from 2017 to 2021 (per 100,000).

Figure S2. The prevalence and mortality rates of stroke by gender in Changwu Town from 2017 to 2021 (per 100,000).

Table S4. The prevalence and mortality rates of stroke by age (years old) in Changwu Town from 2017 to 2021 (per 100,000).

Figure S3. The prevalence and mortality rates of stroke by age in Changwu Town from 2017 to 2021 (per 100,000).

Table S5. The prevalence and mortality rates of stroke by disease types in Changwu Town from 2017 to 2021 (per 100,000).

Figure S4. The prevalence and mortality rates of stroke by disease types in Changwu Town from 2017 to 2021 (per 100,000).

Table S6. The numbers of stroke patients and deaths under different levels of fluoride exposure in Changwu Town from 2017 to 2021.

Table S7. The prevalence and mortality rates of stroke in endemic and non-endemic fluorosis areas in Changwu Town from 2017 to 2021 (per 100,000).

Table S8. The all-ages and age-standardized prevalence rates of stroke by gender in endemic and non-endemic groups in Changwu Town from 2017 to 2021 (per 100,000).

Figure S5. The all-ages and age-standardized prevalence rates of stroke by gender in endemic and non-endemic groups in Changwu Town from 2017 to 2021 (per 100,000).

Table S9. The all-ages and age-standardized mortality rates of stroke by gender in endemic and non-endemic groups in Changwu Town from 2017 to 2021 (per 100,000).

Figure S6. The all-ages and age-standardized mortality rates of stroke by gender in endemic and non-endemic groups in Changwu Town from 2017 to 2021 (per 100,000).

Table S10. The prevalence and mortality rates of stroke in different current water fluoride (CWF) groups in Changwu Town from 2017 to 2021 (per 100,000).

Table S11. The prevalence and mortality rates of stroke in groups with different water improvement period (years) in Changwu Town from 2017 to 2021 (per 100,000).

Table S12. The prevalence and mortality rates of stroke in different WFCEI groups in Changwu Town from 2017 to 2021 (per 100,000).

Abbreviations

APC: annual percentage change

CEDC: Center for Endemic Disease Control

CWF: current water fluoride

WFCEI: water fluoride cumulative exposure index

Table S1. Annual population of permanent residents in Changwu Town from 2017 to 2021.

Year	Total	Sex		Age (years old)					
		Male	Female	≤34	35-44	45-54	55-64	65-74	≥75
2017	22983	12064	10920	4379	3981	4245	4379	3353	2647
2018	22569	11868	10701	4328	3887	4143	4329	3272	2611
2019	22453	11801	10652	4278	3860	4104	4305	3292	2615
2020	22342	11726	10617	4250	3825	4084	4290	3290	2604
2021	22181	11665	10516	4220	3792	4045	4233	3302	2590

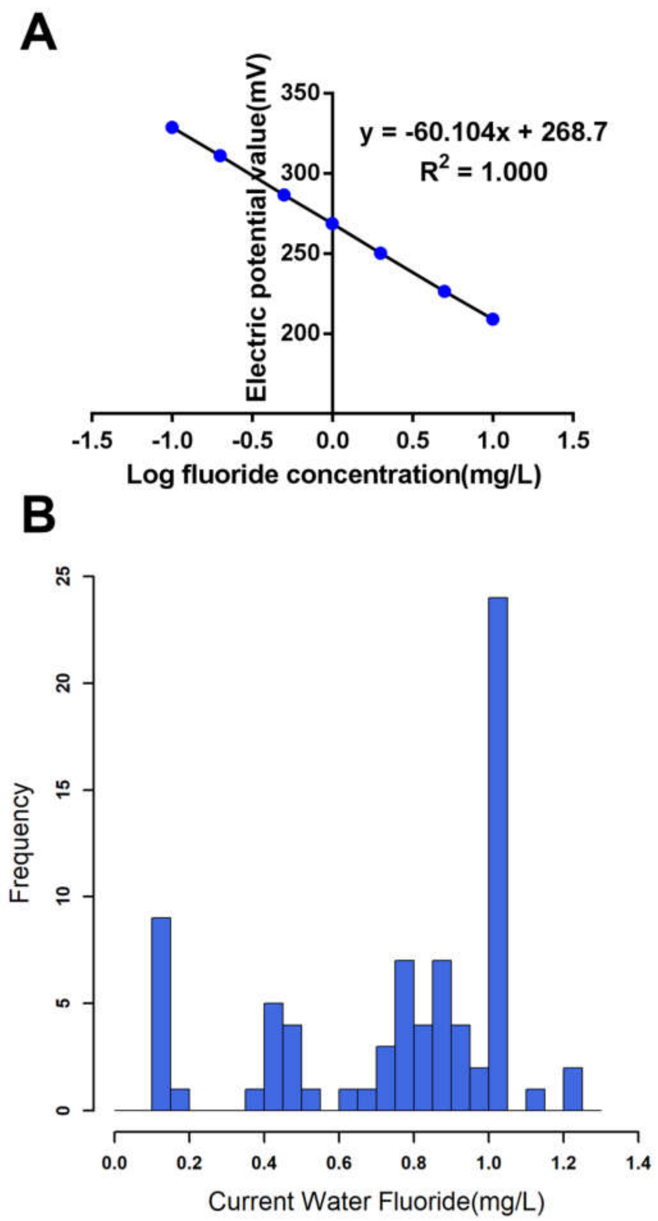

Figure S1. The detection and result of CWF concentration of 77 villages in Changwu Town. (A) The standard curve about the measured electric potential values and the Log concentration of CWF. (B) Histogram of CWF concentration of 77 villages in Changwu Town.

Table S2. The numbers of stroke patients and deaths by sex, age and disease types in Changwu Town from 2017 to 2021.

	Year	Total	Sex		Age (years old)						Disease types	
			Male	Female	≤34	35-44	45-54	55-64	65-74	≥75	Ischemic	Hemorrhagic
Patients	2017	483	277	206	3	27	102	195	116	40	453	40
	2018	633	351	282	3	33	138	254	155	50	597	49
	2019	763	417	346	4	34	162	301	206	56	723	54
	2020	866	470	396	4	39	182	345	245	51	820	61
	2021	978	537	441	4	40	203	385	291	55	928	66
Deaths	2017	27	17	10	0	1	2	3	8	13	18	9
	2018	35	20	15	0	0	3	4	15	13	19	16
	2019	58	30	28	0	2	2	15	17	22	42	16
	2020	60	33	27	0	1	3	8	19	29	46	14
	2021	25	15	10	0	1	2	8	6	8	16	9

Table S3. The prevalence and mortality rates of stroke by gender in Changwu Town from 2017 to 2021 (per 100,000).

Year	All-ages prevalence rate				Age-standardized prevalence rate				All-ages mortality rate				Age-standardized mortality rate			
	Male	Female	Chi-square	*P*-value #	Male	Female	Chi-square	*P*-value #	Male	Female	Chi-square	*P*-value #	Male	Female	Chi-square	*P*-value #
2017	2296.2	1886.5	**4.484***	**0.034**	1571.7	1313.8	2.072	0.150	140.9	91.6	1.186	0.276	73.7	52.8	0.339	0.560
2018	2957.5	2635.3	2.027	0.155	2035.9	1824.1	0.937	0.333	168.5	140.2	0.291	0.590	95.9	79.0	0.047	0.829
2019	3533.7	3248.2	1.298	0.255	2421.0	2243.3	0.486	0.486	254.2	262.9	0.016	0.899	140.1	148.6	0.084	0.772
2020	4008.4	3730.0	1.072	0.300	2750.7	2575.9	0.341	0.560	281.4	254.3	0.152	0.696	156.6	126.2	0.182	0.670
2021	4603.7	4193.6	2.019	0.155	3142.8	2897.4	0.650	0.420	128.6	95.1	0.550	0.458	73.3	56.4	0.330	0.566
APC	**17.9****	**20.2****			**17.8****	**20.0****			7.6	11.8			8.5	8.7		
APC 95%CI	12.7~23.3	11.2~30.0			12.4~23.4	11.3~29.5			-30.5~66.4	-41.8~114.9			-29.9~67.9	-41.0~100.1		
t-value	11.7	7.5			11.1	7.7			0.5	0.5			0.6	0.4		
P-value†	**0.001**	**0.005**			**0.002**	**0.005**			0.631	0.624			0.595	0.694		

Abbreviations: APC, annual percentage change.

P-Value for chi-square comparison between two groups.

† *P*-Value for APC comparison over five years.

P < 0.05 is indicated in bold, * *P*<0.05, ** *P*<0.01, *** *P*<0.001.

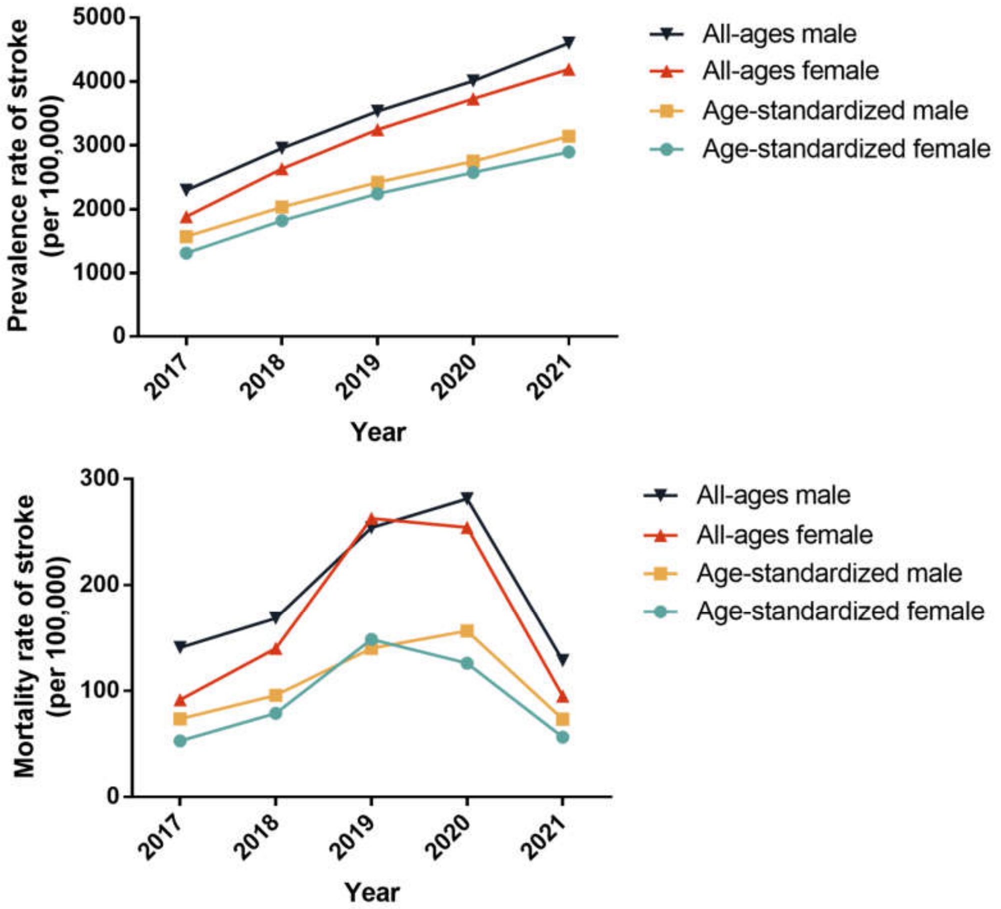

Figure S2. The prevalence and mortality rates of stroke by gender in Changwu Town from 2017 to 2021 (per 100,000).

Table S4. The prevalence and mortality rates of stroke by age (years old) in Changwu Town from 2017 to 2021 (per 100,000).

Year	Prevalence rate								Mortality rate							
	≤34	35-44	45-54	55-64	65-74	≥75	Chi-square	*P*-value#	≤34	35-44	45-54	55-64	65-74	≥75	Chi-square	*P*-value#
2017	68.5	678.3	2402.8	4453.1	3460.1	1511.1	**269.060*** **	**< 0.001**	0.00	25.1	47.1	68.5	238.6	491.1	**32.271*** **	**< 0.001**
2018	69.3	849.1	3330.9	5867.4	4737.2	1915.0	**357.496*** **	**< 0.001**	0.00	0.0	72.4	92.4	458.4	497.9	**37.512*** **	**< 0.001**
2019	93.5	880.8	3947.8	6991.9	6258.5	2141.5	**451.588*** **	**< 0.001**	0.00	51.8	48.7	348.4	516.5	841.3	**44.306*** **	**< 0.001**
2020	94.1	1019.6	4456.4	8042.0	7447.9	1958.5	**542.536*** **	**< 0.001**	0.00	26.1	73.5	186.5	577.6	1113.7	**75.143*** **	**< 0.001**
2021	94.8	1055.0	5019.2	9095.2	8814.2	2123.6	**633.763*** **	**< 0.001**	0.00	26.4	49.4	189.0	181.7	308.9	**12.329***	**0.015**
APC	9.6	**10.9***	**18.2** **	**18.3** **	**24.9** **	6.6					0.8	14.6	3.4	11.3		
APC 95%CI	-0.5~20.7	4.9~17.3	9.8~27.3	12.2~24.7	17.3~33.0	-3.5~17.7					-23.9~33.5	-48.3~154.1	-43.0~87.6	-39.9~106.0		
t-value	3.0	5.9	7.2	10.1	11.3	2.0					0.1	0.5	0.2	0.6		
P-value†	0.057	**0.010**	**0.005**	**0.002**	**0.001**	0.134					0.935	0.623	0.869	0.619		

Abbreviations: APC, annual percentage change.

P-Value for chi-square comparison among six or five groups, the group with 0 cases is not included in the chi-square analysis.

† *P*-Value for APC comparison over five years.

P < 0.05 is indicated in bold, * *P*<0.05, ** *P*<0.01, *** *P*<0.001.

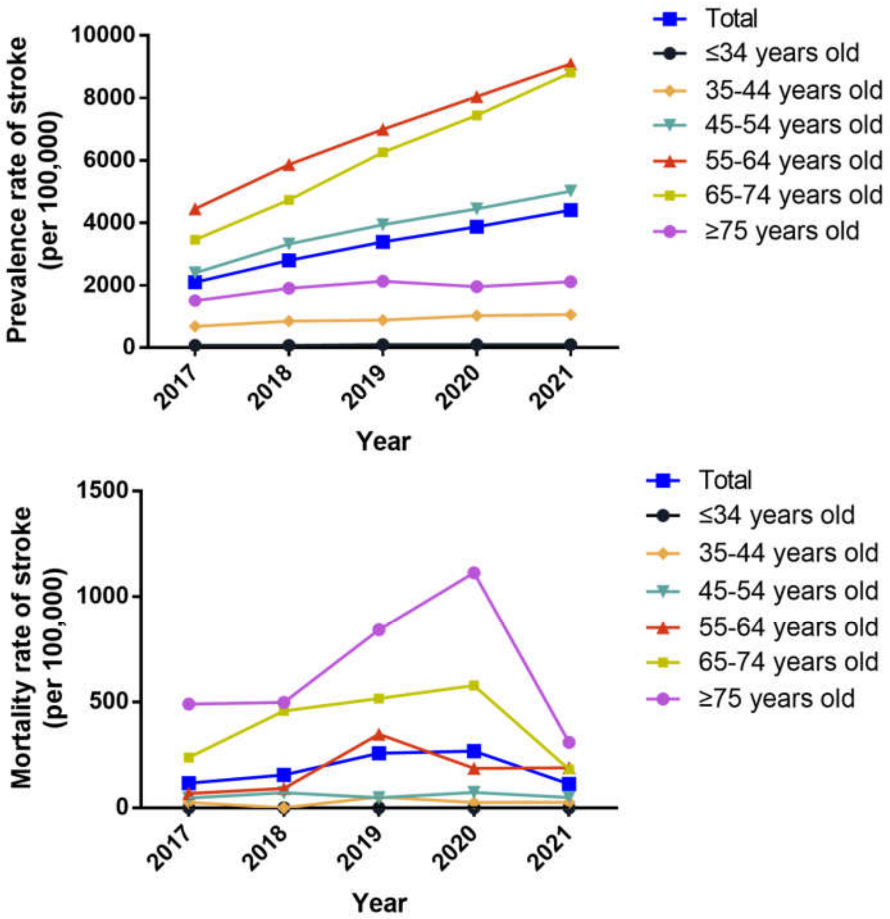

Figure S3. The prevalence and mortality rates of stroke by age in Changwu Town from 2017 to 2021 (per 100,000).

Table S5. The prevalence and mortality rates of stroke by disease types in Changwu Town from 2017 to 2021 (per 100,000).

Year	All-ages prevalence rate				Age-standardized prevalence rate				All-ages mortality rate				Age-standardized mortality rate			
	Ischemic	Hemorrhagic	Chi-square	*P*-value#	Ischemic	Hemorrhagic	Chi-square	*P*-value#	Ischemic	Hemorrhagic	Chi-square	*P*-value#	Ischemic	Hemorrhagic	Chi-square	*P*-value#
2017	1971.0	174.0	**342.337*** **	**＜ 0.001**	1353.4	131.3	**229.860*** **	**＜ 0.001**	78.3	39.2	2.998	0.083	39.9	24.1	1.384	0.239
2018	2645.2	217.1	**458.373*** **	**＜ 0.001**	1817.5	160.4	**310.576*** **	**＜ 0.001**	84.2	70.9	0.257	0.612	44.2	43.2	1.470	0.225
2019	3220.1	240.5	**566.336*** **	**＜ 0.001**	2207.0	175.3	**385.815*** **	**＜ 0.001**	187.1	71.3	**11.642** **	**0.001**	100.5	43.1	**5.117***	**0.024**
2020	3670.2	273.0	**641.429*** **	**＜ 0.001**	2518.3	198.1	**437.868*** **	**＜ 0.001**	205.9	62.7	**17.047*** **	**＜ 0.001**	106.9	35.7	**7.994** **	**0.005**
2021	4183.9	297.6	**731.411*** **	**＜ 0.001**	2863.5	214.7	**496.928*** **	**＜ 0.001**	72.1	40.6	1.959	0.162	39.3	26.6	0.053	0.819
APC	**19.2** **	**13.50** **			**19.1** **	**12.4** **			15.7	-1.3			14.7	-2.3		
APC 95%CI	12.1~26.8	8.90~18.4			12.0~26.7	8.4~16..6			-39.6~121.4	-30.6~40.5			-39.6~117.7	-29.7~35.8		
t-value	9.1	9.6			9.0	10.3			0.7	-0.1			0.7	-0.2		
P-value†	**0.003**	**0.002**			**0.003**	**0.002**			0.527	0.916			0.544	0.835		

Abbreviations: APC, annual percentage change.

P-Value for chi-square comparison between two groups.

† *P*-Value for APC comparison over five years.

P < 0.05 is indicated in bold, * *P*<0.05, ** *P*<0.01, *** *P*<0.001.

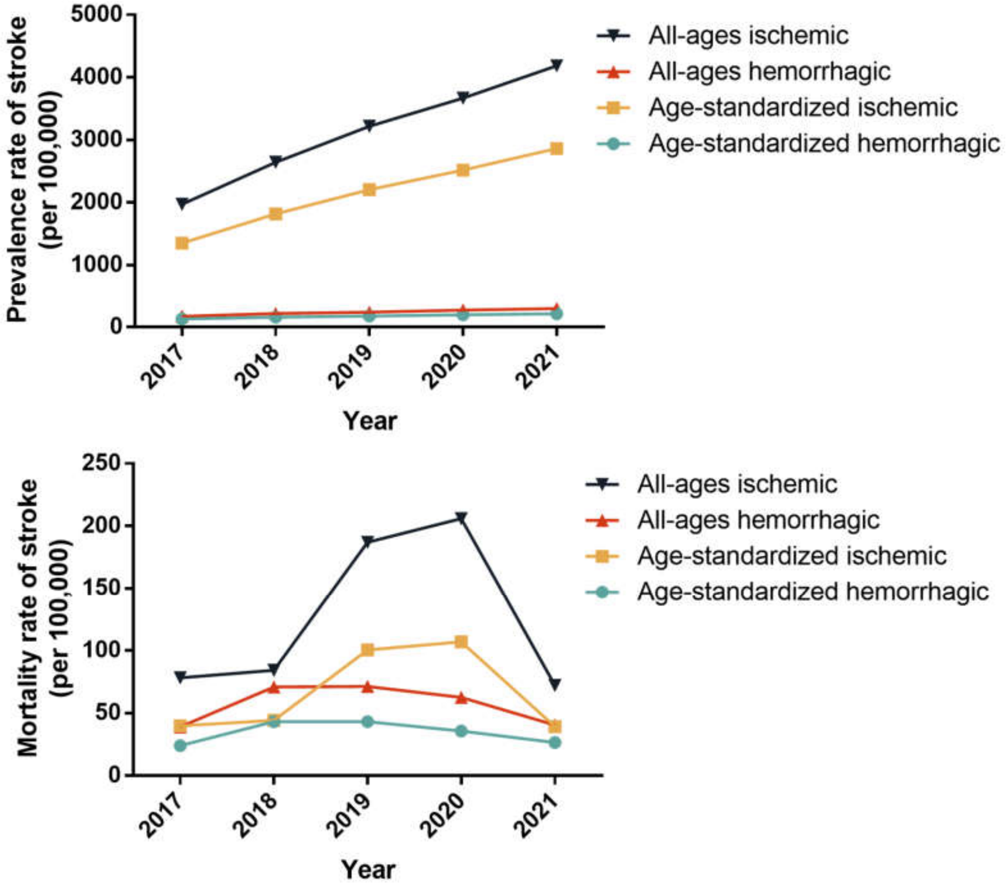

Figure S4. The prevalence and mortality rates of stroke by disease types in Changwu Town from 2017 to 2021 (per 100,000).

Table S6. The numbers of stroke patients and deaths under different levels of fluoride exposure in Changwu Town from 2017 to 2021.

	Year	Total	Historical water fluoride		CWF		Water improvement period					WFCEI#		
			Endemic (> 1.2 mg/L)	Non-endemic(≤ 1.2 mg/L)	≤ 1 mg/L	> 1 mg/L	Non-endemic areas	1~5 years	6~10 years	11~15 years	16~20 years	Tertile 1	Tertile 2	Tertile 3
Annual population	2017	22983	10503	12480	12137	10847	12480	0	4478	3744	2282	11746	4557	6123
	2018	22569	10365	12204	11845	10725	12204	0	4438	3680	2248	11474	4544	6019
	2019	22453	10374	12079	11789	10664	12079	0	4437	3689	2249	11354	4588	5992
	2020	22342	10345	11998	11723	10620	11998	0	4421	3685	2239	11277	4576	5982
	2021	22181	10356	11825	11631	10550	11825	0	4426	3724	2207	11113	4580	6010
Patients	2017	483	321	162	380	103	162	0	140	70	111	162	126	187
	2018	633	428	205	500	133	205	0	192	100	136	209	166	250
	2019	763	519	244	602	161	244	0	238	119	162	242	212	300
	2020	866	573	293	677	189	293	0	256	141	176	284	237	331
	2021	978	622	356	744	234	356	0	270	162	190	353	249	361
Deaths	2017	27	11	16	13	14	16	0	2	7	2	15	6	6
	2018	35	18	17	19	16	17	0	5	10	3	11	13	11
	2019	58	26	32	31	27	32	0	5	18	3	32	7	19
	2020	60	28	32	28	32	32	0	13	7	8	29	11	20
	2021	25	9	16	12	13	16	0	5	3	1	15	6	4

Abbreviations: CWF, current water fluoride concentration; WFCEI, water fluoride cumulative exposure index.

All participants were divided into three groups based on tertile ranges of their WFCEI (Tertile 1 group: 7.00-51.00; Tertile 2 group: 51.53-74.61; Tertile 3 group: 74.93-199.38).

Table S7. The prevalence and mortality rates of stroke in endemic and non-endemic fluorosis areas in Changwu Town from 2017 to 2021 (per 100,000).

Year	All-ages prevalence rate				Age-standardized prevalence rate				All-ages mortality rate				Age-standardized mortality rate			
	Endemic	Non-endemic	Chi-square	*P*-value#	Endemic	Non-endemic	Chi-square	P-value#	Endemic	Non-endemic	Chi-square	P-value#	Endemic	Non-endemic	Chi-square	P-value#
2017	3056.3	1298.1	**82.048*****	**<0.001**	2184.1	829.9	**70.322*****	**<0.001**	104.7	128.2	0.267	0.605	63.4	65.9	0.006	0.940
2018	4129.3	1679.8	**116.421*****	**<0.001**	2935.0	1094.5	**95.257*****	**<0.001**	173.7	139.3	0.426	0.514	106.1	76.5	<0.001	0.993
2019	5002.9	2020.1	**141.041*****	**<0.001**	3514.5	1342.5	**109.964*****	**<0.001**	250.6	264.9	0.044	0.834	153.7	137.1	0.003	0.954
2020	5539.2	2442.2	**132.063*****	**<0.001**	3881.0	1641.8	**100.766*****	**<0.001**	270.7	266.7	0.003	0.955	164.1	126.8	0.002	0.965
2021	6006.2	3010.7	**107.436*****	**<0.001**	4203.8	2020.0	**83.640*****	**<0.001**	86.9	135.3	1.146	0.284	56.9	72.6	0.001	0.969
APC	**17.3****	**22.1*****			**16.2****	**24.1*****			8.2	10.9			9.4	7.6		
APC 95%CI	8.1~27.4	19.6~24.7			7.3~25.9	21.1~27.1			-41.9~101.6	-30.1~76.2			-39.5~97.5	-30.2~65.9		
t-value	6.2	30.3			6.0	28.7			0.4	0.7			0.5	0.5		
P-value†	**0.008**	**<0.001**			**0.009**	**<0.001**			0.714	0.527			0.663	0.628		

Note: APC, annual percentage change.

# P-Value for chi-square comparison between two groups.

† P-Value for APC comparison over five years.

P < 0.05 is indicated in bold, * *P*<0.05, ** *P*<0.01, *** *P*<0.001..

Table S8. The all-ages and age-standardized prevalence rates of stroke by gender in endemic and non-endemic groups in Changwu Town from 2017 to 2021 (per 100,000).

Year	All-ages prevalence rate								Age-standardized prevalence rate							
	Male				Female				Male				Female			
	Endemic	Non-endemic	Chi-square	*P*-value#	Endemic	Non-endemic	Chi-square	*P*-value#	Endemic	Non-endemic	Chi-square	*P*-value#	Endemic	Non-endemic	Chi-square	*P*-value#
2017	3445.4	1383.3	53.747***	**< 0.001**	2653.8	1198.5	29.957***	**< 0.001**	2405.4	893.1	42.477***	**< 0.001**	1944.7	758.7	27.994***	**< 0.001**
2018	4478.2	1743.0	71.784***	**< 0.001**	3768.4	1605.4	46.128***	**< 0.001**	3108.4	1158.0	54.447***	**< 0.001**	2751.5	1026.9	41.539***	**< 0.001**
2019	5339.4	2070.9	84.919***	**< 0.001**	4653.9	1960.6	57.431***	**< 0.001**	3631.4	1426.0	57.410***	**< 0.001**	3389.2	1249.3	53.131***	**< 0.001**
2020	5961.1	2415.6	87.179***	**< 0.001**	5101.4	2473.1	47.264***	**< 0.001**	4039.9	1692.1	56.927***	**< 0.001**	3708.5	1590.0	44.376***	**< 0.001**
2021	6490.7	3040.8	71.217***	**< 0.001**	5501.3	29755	38.310***	**< 0.001**	4403.8	2098.3	47.370***	**< 0.001**	3990.9	1940.0	36.087***	**< 0.001**
APC	15.9**	20.1***			17.6*	24.8***			15.1**	22.9***			17.3*	25.7***		
APC 95%CI	8.7~23.6	17.9~24.2			5.9~30.5	20.8~28.8			8.4~22.2	19.8~26.1			5.8~30.1	21.9~29.6		
t-value	7.3	23.3			4.9	22.0			7.5	25.8			4.9	24.0		
P-value†	**0.005**	**<0.001**			**0.016**	**<0.001**			**0.005**	**<0.001**			**0.016**	**<0.001**		

Abbreviations: APC, annual percentage change.

P-Value for chi-square comparison between two groups.

† *P*-Value for APC comparison over five years.

$P < 0.05$ is indicated in bold, * P<0.05, ** P<0.01, *** P<0.001.

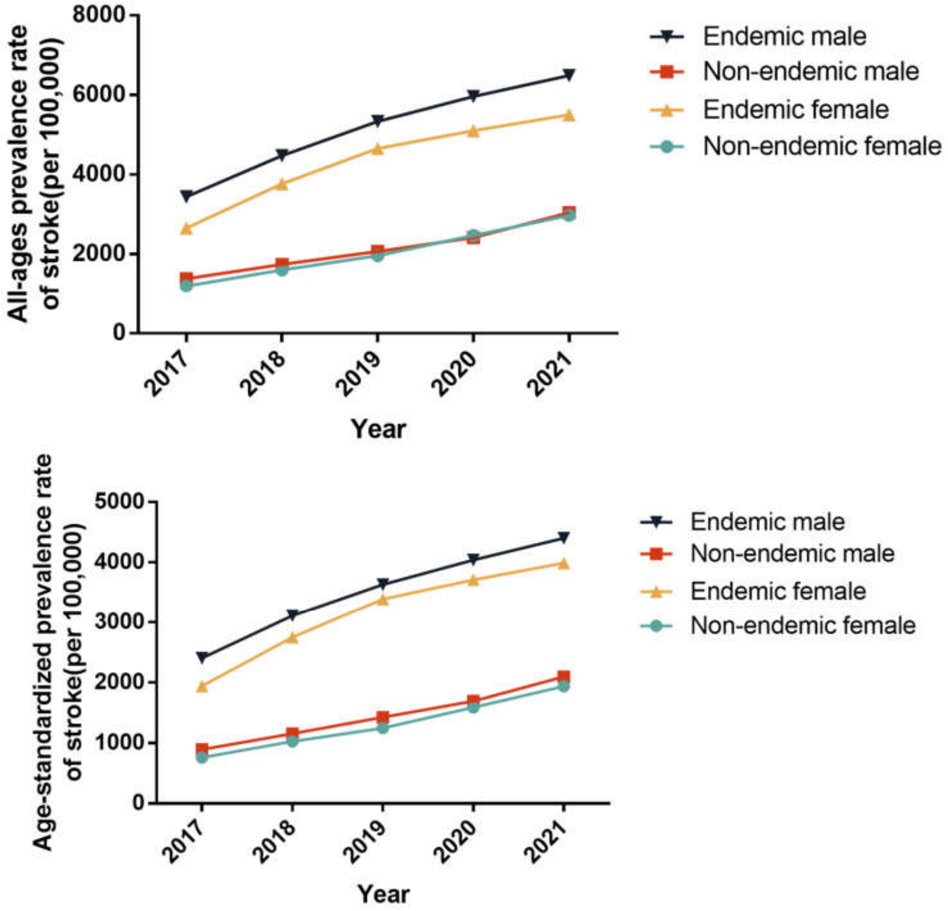

Figure S5. The all-ages and age-standardized prevalence rates of stroke by gender in endemic and non-endemic groups in Changwu Town from 2017 to 2021 (per 100,000).

Table S9. The all-ages and age-standardized mortality rates of stroke by gender in endemic and non-endemic groups in Changwu Town from 2017 to 2021 (per 100,000).

Year	All-ages mortality rate								Age-standardized mortality rate							
	Male				Female				Male				Female			
	Endemic	Non-endemic	Chi-square	*P*-value#	Endemic	Non-endemic	Chi-square	*P*-value#	Endemic	Non-endemic	Chi-square	*P*-value#	Endemic	Non-endemic	Chi-square	*P*-value#
2017	149.8	133.9	0.053	0.817	58.1	121.6	1.197	0.274	87.1	65.5	0.464	0.496	37.5	64.8	0.468	0.494
2018	189.6	151.6	0.253	0.615	157.0	124.9	0.197	0.658	111.2	89.0	0.152	0.697	101.3	62.9	0.227	0.633
2019	208.3	291.5	0.793	0.373	294.6	233.8	0.372	0.542	124.4	148.7	0.088	0.767	187.5	124.9	0.825	0.364
2020	265.8	294.2	0.083	0.773	275.8	234.7	0.175	0.675	161.6	152.5	0.046	0.831	167.7	96.8	1.519	0.218
2021	113.5	141.1	0.170	0.680	59.2	128.6	1.329	0.249	70.7	77.4	0.003	0.959	41.4	65.6	0.533	0.465
APC	3.4	11.1			15.2	9.3			3.6	10.8			14.7	4.7		
APC 95%CI	-30.0~53.0	-32.6~83.0			-55.2~196.3	-28.3~66.5			-30.5~54.5	-31.1~78.1			-54.4~188.4	-30.2~57.0		
t-value	0.3	0.7			0.5	0.7			0.3	0.7			0.5	0.4		
P-value†	0.801	0.551			0.665	0.550			0.797	0.542			0.668	0.742		

Abbreviations: APC, annual percentage change.

P-Value for chi-square comparison between two groups.

† *P*-Value for APC comparison over five years.

P < 0.05 is indicated in bold, * *P*<0.05, ** *P*<0.01, *** *P*<0.001.

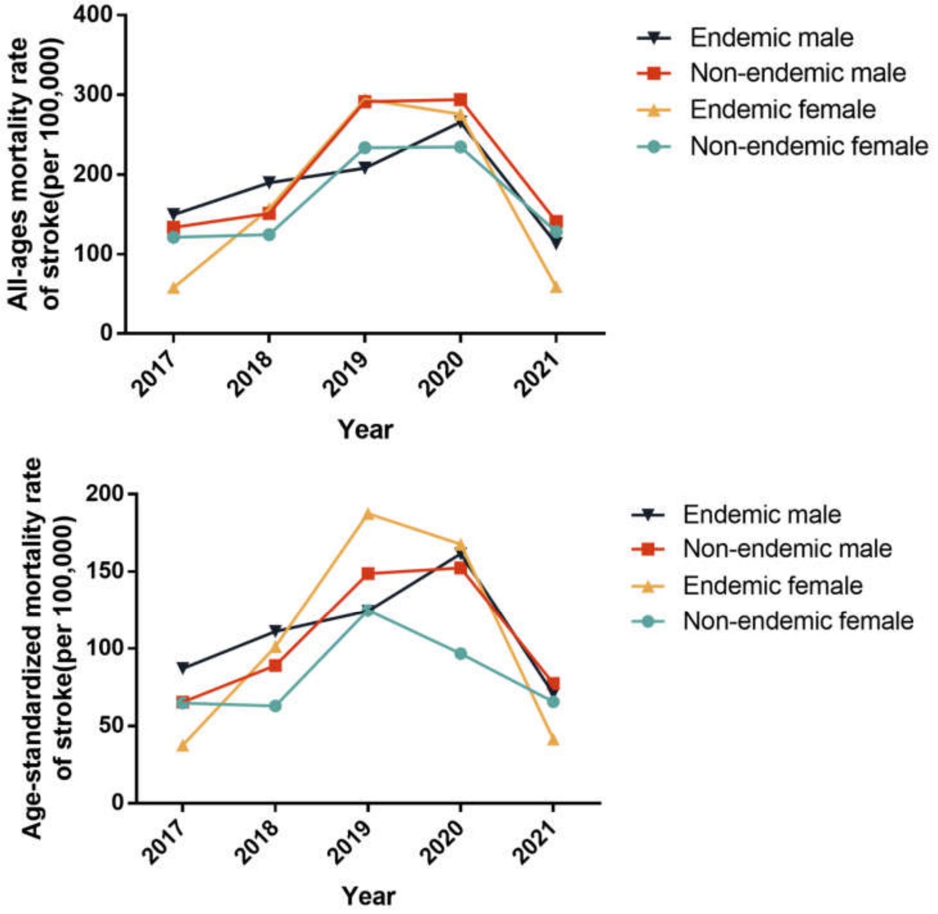

Figure S6. The all-ages and age-standardized mortality rates of stroke by gender in endemic and non-endemic groups in Changwu Town from 2017 to 2021 (per 100,000).

Table S10. The prevalence and mortality rates of stroke in different current water fluoride (CWF) groups in Changwu Town from 2017 to 2021 (per 100,000).

Year	All-ages prevalence rate				Age-standardized prevalence rate				All-ages mortality rate				Age-standardized mortality rate			
	CWF ≤ 1 mg/L	CWF > 1 mg/L	Chi-square	*P*-value#	CWF ≤ 1 mg/L	CWF > 1 mg/L	Chi-square	*P*-value#	CWF ≤ 1 mg/L	CWF > 1 mg/L	Chi-square	*P*-value#	CWF ≤ 1 mg/L	CWF > 1 mg/L	Chi-square	*P*-value#
2017	3131.1	949.6	**127.193*****	**< 0.001**	2129.7	636.2	**88.159*****	**< 0.001**	107.1	129.1	0.235	0.628	59.8	68.6	0.044	0.833
2018	4221.4	1240.2	**173.774*****	**< 0.001**	2868.4	839.5	**119.748*****	**< 0.001**	160.4	149.2	0.046	0.831	93.8	83.0	0.051	0.822
2019	5106.5	1509.8	**206.565*****	**< 0.001**	3448.4	1024.3	**140.850*****	**< 0.001**	263.0	253.2	0.021	0.886	151.3	136.0	0.055	0.815
2020	5775.2	1779.7	**221.420*****	**< 0.001**	3890.6	1233.1	**146.027*****	**< 0.001**	238.9	301.3	0.808	0.369	137.8	146.4	0.078	0.780
2021	6397.0	2218.0	**210.326*****	**< 0.001**	4293.6	1540.2	**136.997*****	**< 0.001**	103.2	123.2	0.197	0.657	64.3	69.4	0.033	0.855
APC	**18.0****	**22.6*****			**17.6****	**23.70*****			6.7	11.9			6.1	9.4		
APC 95%CI	9.9~26.8	19.2~26.1			9.5~26.4	20.60~26.9			-38.7~85.7	-33.1~87.2			-38.5~82.9	-35.1~84.3		
t-value	7.4	23.0			7.2	26.3			0.4	0.7			0.3	0.5		
P-value†	**0.005**	**<0.001**			**0.006**	**<0.001**			0.734	0.536			0.753	0.624		

Abbreviations: APC, annual percentage change; CWF, current water fluoride.

\# *P*-Value for chi-square comparison between two groups.

† *P*-Value for APC comparison over five years.

P < 0.05 is indicated in bold, * *P*<0.05, ** *P*<0.01, *** *P*<0.001.

Table S11. The prevalence and mortality rates of stroke in groups with different water improvement period (years) in Changwu Town from 2017 to 2021 (per 100,000).

Year	All-ages prevalence rate						Age-standardized prevalence rate						All-ages mortality rate						Age-standardized mortality rate					
	Non-endemic areas	6~10	11~15	16~20	Chi-square	*P*-value#	Non-endemic areas	6~10	11~15	16~20	Chi-square	*P*-value#	Non-endemic areas	6~10	11~15	16~20	Chi-square	*P*-value#	Non-endemic areas	6~10	11~15	16~20	Chi-square	*P*-value#
2017	1298.1	3126.7	1869.9	4864.2	**139.522*** **	**< 0.001**	829.9	2293.8	1280.1	3825.1	**135.924*** **	**< 0.001**	128.2	44.7	187.0	87.6	3.851	0.278	65.9	29.8	148.7	38.4	6.122	0.106
2018	1679.8	4326.8	2717.4	6051.2	**168.962*** **	**< 0.001**	1094.5	3154.5	1867.1	4696.4	**157.999*** **	**< 0.001**	139.3	112.7	271.7	133.5	3.999	0.262	76.5	70.7	218.2	66.2	7.468	0.058
2019	2020.1	5364.6	3226.2	7203.2	**203.764*** **	**< 0.001**	1342.5	3851.0	2188.7	5470.0	**178.219*** **	**< 0.001**	264.9	112.7	488.0	133.4	**12.510** **	**0.006**	137.1	61.3	344.0	65.8	**13.652** **	**0.003**
2020	2442.2	5790.5	3826.3	7862.4	**186.709*** **	**< 0.001**	1641.8	4141.9	2565.8	5911.3	**160.712*** **	**< 0.001**	266.7	294.1	190.0	357.4	1.609	0.657	126.8	162.1	166.1	202.5	1.373	0.712
2021	3010.7	6101.0	4350.2	8610.9	**159.310*** **	**< 0.001**	2020.0	4341.9	2958.3	6421.2	**139.954*** **	**< 0.001**	135.3	113.0	80.6	45.3	1.765	0.622	72.6	85.9	68.4	20.5	1.859	0.602
APC	**20.2***	**16.2***	**21.1** **	**14.5** **			**21.1***	**15.3***	**20.80** **	**13.00** **			-30.1	32.8	-6.2	31.5			-27.7	31.9	-6.9	46.1		
APC 95% CI	25.1~32.0	4.3~29.5	12.0~30.9	8.4~20.9			27.0~28.6	3.8~28.2	11.8~30.4	7.4~18.9			76.0~0.7	-37.7~182.9	-60.7~123.5	-47.5~229.3			65.6~0.7	-27.0~138.4	-55.1~93.3	-45.3~290.5		
t-value	4.4	4.4	7.8	7.9			4.3	4.3	7.8	7.7			1.2	1.2	-0.2	0.9			1.5	1.5	-0.3	1.2		
P-value†	**0.022**	**0.022**	**0.004**	**0.004**			**0.023**	**0.023**	**0.004**	**0.005**			0.318	0.318	0.828	0.412			0.233	0.233	0.777	0.307		

Abbreviations: APC, annual percentage change.

P-Value for chi-square comparison among four groups.

† *P*-Value for APC comparison over five years.

$P < 0.05$ is indicated in bold, * $P<0.05$, ** $P<0.01$, *** $P<0.001$.

Table S12. The prevalence and mortality rates of stroke in different WFCEI groups in Changwu Town from 2017 to 2021 (per 100,000).

Year	All-ages prevalence rate					Age-standardized prevalence rate					All-ages mortality rate					Age-standardized mortality rate				
	Tertile 1	Tertile 2	Tertile 3	Chi-square	*P*-value#	Tertile 1	Tertile 2	Tertile 3	Chi-square	*P*-value#	Tertile 1	Tertile 2	Tertile 3	Chi-square	*P*-value#	Tertile 1	Tertile 2	Tertile 3	Chi-square	*P*-value#
2017	1379.3	2765.3	3054.3	**63.200*****	**＜0.001**	896.0	2001.8	2211.4	**56.498*****	**＜0.001**	127.7	131.7	98.0	0.355	0.837	61.7	79.2	78.3	0.491	0.782
2018	1821.6	3653.6	4153.9	**86.584*****	**＜0.001**	1201.2	2629.2	3002.9	**74.577*****	**＜0.001**	95.9	286.1	182.8	7.698	0.021	58.1	132.9	110.0	2.403	0.301
2019	2131.4	4620.7	5006.7	**113.902*****	**＜0.001**	1438.9	3249.0	3559.5	**89.051*****	**＜0.001**	281.8	152.6	317.1	2.924	0.232	145.3	72.3	222.9	4.126	0.127
2020	2518.5	5179.2	5533.3	**110.811*****	**＜0.001**	1721.7	3609.7	3924.1	**84.364*****	**＜0.001**	257.2	240.4	334.3	1.093	0.579	123.1	125.8	230.7	3.249	0.197
2021	3176.6	5437.3	6006.7	**79.835*****	**＜0.001**	2166.9	3779.0	4259.3	**62.201*****	**＜0.001**	135.0	131.0	66.6	1.710	0.425	75.0	64.8	102.2	0.508	0.776
APC	**21.9*****	**17.3***	**16.6***			**23.4*****	**16.1***	**16.0***			12.3	-3.7	15.6			11.5	-3.4	15.0		
APC 95%CI	17.7~26.2	6.8~28.8	7.3~26.8			29.4~27.5	6.2~26.9	6.9~25.9			-35.5~95.6	-39.4~53.1	-49.5~164.8			-33.0~85.6	-35.4~44.5	-37.1~110.4		
t-value	18.0	5.4	5.8			20.3	5.3	5.8			0.7	-0.3	0.6			0.7	-0.3	0.7		
P-value†	**<0.001**	**0.012**	**0.010**			**<0.001**	**0.013**	**0.010**			0.552	0.813	0.616			0.546	0.804	0.515		

Note: APC, annual percentage change; WFCEI, water fluoride cumulative exposure index.

# P-Value for chi-square comparison among three groups.

† P-Value for APC comparison over five years.

P < 0.05 is indicated in bold, * *P*<0.05, ** *P*<0.01, *** *P*<0.001.
